# Inner Nuclear Layer Microcyst Configuration, Distribution, and Visual Prognosis in Patients With Epiretinal Membrane After Vitrectomy and Membrane Peeling

**DOI:** 10.1038/s41598-019-48097-1

**Published:** 2019-08-09

**Authors:** Ming-Hung Hsieh, Yu-Bai Chou, Yi-Ming Huang, De-Kuang Hwang, Fang-Yi Tsai, Shih-Jen Chen

**Affiliations:** 1 Department of Ophthalmology, Taipei City Hospital, Taiwan, No.33, Sec. 2, Zhonghua Rd., Zhongzheng Dist., Taipei 10065 Taiwan; 2 Department of Ophthalmology, Taipei Veterans General Hospital, Taiwan, No.201, Shih-Pai Rd., Sec. 2, Taipei, 11217 Taiwan; 3 School of Medicine, National Yang-Ming University, Taiwan, No.155, Sec. 2, Linong St., Beitou Dist., Taipei 11221 Taiwan; 4 Department of Ophthalmology, Taipei Municipal Wanfang Hospital, Taipei, Taiwan No. 111, Sec. 3, Xinglong Rd., Wenshan Dist., Taipei 11696 Taiwan

**Keywords:** Prognostic markers, Retina

## Abstract

Inner nuclear layer(INL) microcysts at central macula are a common finding in patients with epiretinal membrane (ERM) after vitrectomy and membrane peeling. Using en face mode of optical coherence tomography (OCT) angiography, patients with ERM after surgery were retrospectively reviewed to understand the configuration and distribution of microcysts as well as their impact on visual acuity. Forty-six eligible patients were enrolled and their baseline best-corrected visual acuities improved from 20/67 to 20/29 *(P* < 0.01) after surgery. Twenty-eight (60.9%) patients had microcysts that appeared at a median of 5 months after the surgery and persisted for mean 16 months follow-up. The microcyst appeared as spheroidal shape with length ranged from 20 to 80 μm and widths of 80 μm in average. They tend to group in cluster with a density of 245 microcysts per mm^2^. The frequency of microcyst distribution was 86%, 54%, 32%, 25% and 18% at the nasal, superior, inferior, temporal quadrants and central 1 mm, respectively. Linear regression analysis showed that INL microcysts at central and temporal quadrants were associated with poorer visual acuity *(P* = 0.02 and *P* = 0.01, respectively). The presence of INL microcysts in center subfield and involved wider area is a poor prognostic factor for visual outcomes.

## Introduction

Vitrectomy and peeling of the epiretinal membrane (ERM) have been a safe, effective, and widely used treatment strategy for idiopathic ERM^1^. However, some reports have revealed that surgery can cause inner retinal damage, including swelling of the arcuate nerve fiber layer^2^, dissociated optic nerve fiber layer defect^3^, secondary paracentral macular hole^4^, and microcysts in the inner nuclear layer (INL) of the retina^5^.

Studies have reported that the incidence of postoperative INL microcysts is approximately 1–29%^5,6^. The microcysts occurred in patients who presented with more acute symptom onsets, more severe grades of ERM with paravascular anomalies, and intraoperative petechiae after ERM peeling^5^. The INL microcysts appeared as early as 1 month after the surgery, and they may persist for more than 12 months. Although patients with postoperative INL microcysts had improved visual acuity after surgery, the visual gain was less compared with patients without the postoperative microcysts.

Although INL microcysts can be observed with cross-sectional optical coherence tomography (OCT), their distribution, area, and impact on visual prognosis are still unknown. The split-spectrum amplitude-decorrelation angiography OCT with motion correction introduced in 2012 can provide images not only of blood vasculature, but also enhanced 3-dimensional retinal structures by increasing scanning speeds and reducing motion artifacts. The goal of this study was to analyze the characteristics and functional outcomes of INL microcysts after double membrane peeling in patients with ERM using the newer en-face mode OCT.

## Results

### INL microcysts and visual outcome

Forty-six eligible participants (13 males; 33 females; mean age = 64.0 ± 7.2) were enrolled. Their baseline BCVA improved from 20/67 (LogMAR 0.60 ± 0.27) to 20/29 (LogMAR 0.23 ± 0.28) *(P < *0.01) after surgery with a mean follow-up of 16.3 ± 6.5 months. Twenty-eight (60.9%) patients had INL microcysts that appeared at a median of 5 months (1–16 months) after the surgery and persisted during the follow-up period. The distribution of the appearance time of INL microcysts was highly left skewed, with 32% of the patients having microcysts found 3 months after the surgery.

Compared with the nonmicrocystic group, there was a trend of worse preoperative visual acuity and thicker preoperative central macular thickness in the microcystic group. However, there were still no significant differences between postoperative visual acuity and central macular thickness between patients with or without microcysts (Table 1). Furthermore, the duration of symptoms before the operation and the follow-up period after the surgery were not significantly different between the two groups.

**Table 1:** Demographic parameters of the nonmicrocystic group and microcystic group in the inner nuclear layer of patients with idiopathic epiretinal membrane after vitrectomy.

		Nonmicrocystic group		Microcystic group		*p*
		N = 18		N = 28		
Gender	female	12	(67%)	21	(75%)	0.55
	male	6	(33%)	7	(25%)	
Age (y)		64 ± 8		64 ± 6		0.74
Pre-OP BCVA (LogMAR)		0.55 ± 0.24		0.63 ± 0.28		0.32
Post-OP BCVA (LogMAR)		0.16 ± 0.16		0.27 ± 0.33		0.12
Pre-OP CMT (um)		454 ± 156		506 ± 77		0.14
Post-OP CMT (um)		359 ± 32		376 ± 44		0.15
Symptom duration (m)		16 ± 11		18 ± 17		0.65
Follow-up duration (m)		16 ± 7		16 ± 7		0.89
Final lens status						0.71
	pseudophakia	14	(78%)	24	(86%)	
	clear lens	3	(17%)	2	(7%)	
	cataract	1	(5%)	2	(7%)	

Abbreviation: CMT, central macular thickness.

### Morphology and distribution of INL microcysts

**Figure 1:**
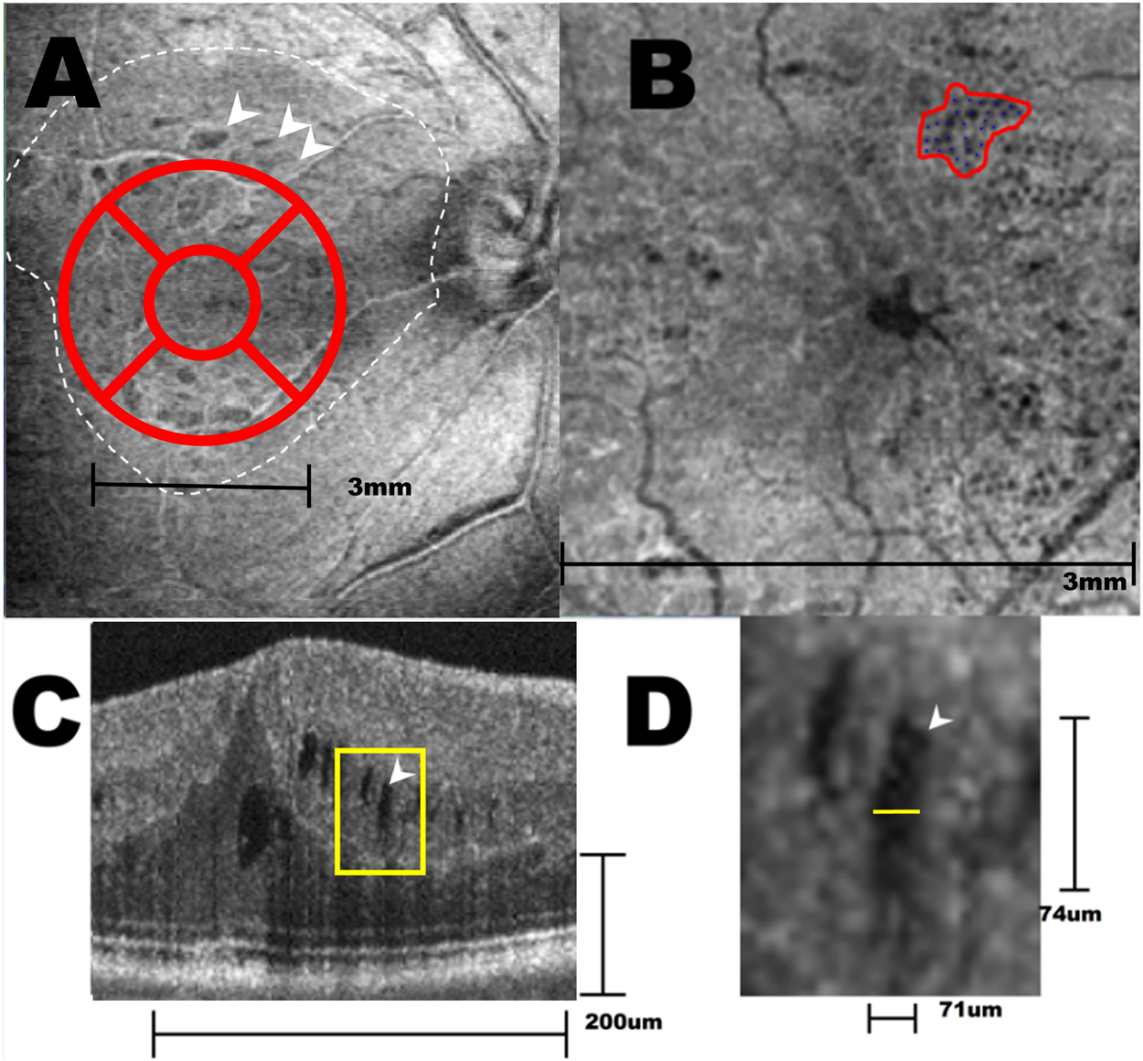
En face OCT and cross sectional OCT of a right eye that had postoperative microcysts. (**A**) The central 3 × 3 mm macula was divided into 5 Early Treatment for Diabetic Retinopathy Study (ETDRS) areas in this en face image at the superficial capillary plexus level. The center subfield was 1 mm in diameter. The white dotted line indicates the area of ILM being peeled. White arrows indicate dissociated optic nerve fiber layer within the area of denuded ILM. (**B**) En face view at the deep capillary plexus of the same eye in center 3 × 3 mm of (**A**) showed microcystic formations in the nasal subfield that extend to the superior and inferior subfields. The area of each cluster and their density of microcyst were calculated as in the irregular circle. (**C**) In this cross sectional OCT, the microcysts appeared as multiple spindle shaped hollow cavity in the inner nuclear layer. Note that the vertical and horizontal scale lines represent 200 μm. The yellow rectangle area was magnified as shown in (**D**). (**D**) The width and length of microcysts determined by a built-in function of the Avanti RTVue XR show that the “spindle-shaped” microcyst actually approximates a spheroid shape (71 × 74 μm) after adjusting the vertical and horizontal scales.

We measured the vertical distance of INL microcyst from top to down as the length in B-scan of the OCT, and the maximum distance from side to side as the width in cross-sectional OCT image. The length of the individual INL microcysts ranged from 20 to 80 μm, and the width ranged from 60 to 120 μm, with 80 μm in average. We also measured the maximum diameter of the microcysts in the en face view, and found that it ranged from 32 to 192 μm, with 72 μm in average. This is because that the composite of multiple A scan lines in the en face mode could pick up smaller circular microcysts than the cross sectional scans. These observations suggest that the INL microcyst is actually a spheroid space that has been vertically distorted into a spindle shape in the conventional reporting of commercial OCT because of uncoordinated horizontal and vertical scales (Fig. 1). The microcysts generally grouped together in a clustered honeycomb pattern with many clustering near the paramacular area. For each microcyst cluster, the density was approximately 245 microcysts per mm^2^ (Fig. 1).

In the subfield analysis in Table 2, 86% of the patients who had microcysts had microcysts involving the nasal subfield. Other involved subfields with microcysts were 18% at the center 1 mm in diameter, and 54%, 32%, and 25% at the superior, inferior, and temporal quadrants, respectively. Regarding the numbers of involved areas, 11 patients had INL microcysts in only one subfield; 7, 6, and 4 patients had microcysts in 2; and 3 had microcysts in more than 3 subfields, respectively. Among the 11 patients who had microcysts in 1 subfield, 8 had microcysts only at the nasal field, whereas 3 developed them at the center subfield. For those 10 patients who had at least 3 or more subfields affected, all involved the nasal and superior subfields, 8 involved the inferior subfield, 6 involved the temporal subfield, and only 1 involved the center subfield.

**Table 2:** Distribution of inner nuclear layer microcysts in 28 patients with microcysts after vitrectomy.

Area	N	%
Central	5	18
Superior	15	54
Nasal	24	86
Inferior	9	32
Temporal	7	25

**Figure 2:**
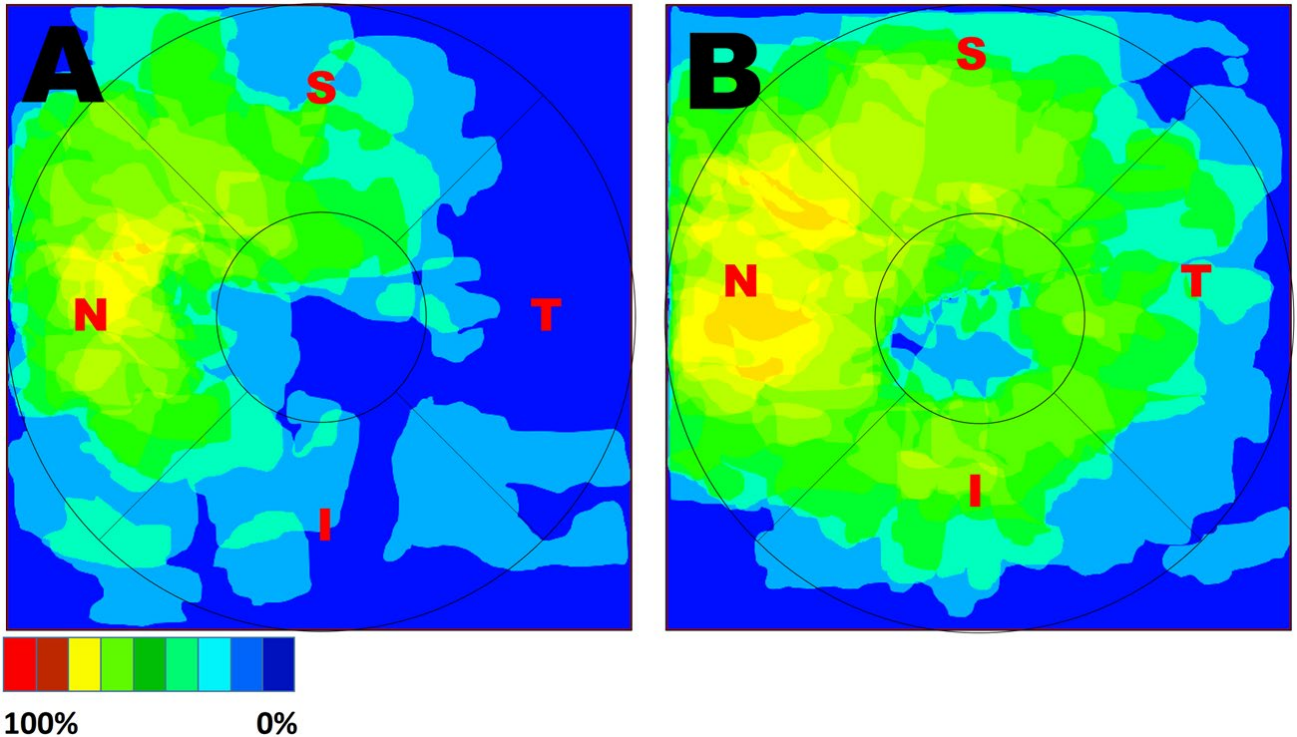
The distribution and frequency map of inner nuclear layer (INL) microcysts of 28 patients according to their final visual acuity. (**A**) greater than or equal to 20/29 (*N* = 15) and (**B**) less than 20/29 (*N* = 13). In the poor visual acuity subgroup (**B**), the microcysts involved a larger area and were more frequent in the central, temporal, and inferior areas. Both groups had microcysts that were mostly involved in the nasal subfield.

Patients who had a final visual acuity of less than 20/29 had larger areas of INL microcysts that covered temporal, inferior, and central areas in addition to the nasal subfield (Fig. 2).

### Factors associated with visual outcome

Linear regression analysis showed that, among patients with microcysts, those who had worse final visual acuities had microcysts at the central and temporal quadrants *(P* = 0.02, *P* = 0.01, respectively) (Table 3). Numbers of affected subfields of 4 or more were associated with worse visual acuity *(P* < 0.01) compared with those who had less than 3 subfields affected.

**Table 3:** Regression analysis of the association between the presence of microcysts and postoperative visual acuity in each area and number of subfield^*^.

Affected subfield	Post-OP BCVA (LogMAR)		
	beta	95%CI	p
Central	0.227	0.034~0.420	0.022
Superior	−0.008	0.153~0.136	0.906
Nasal	0.021	0.103~0.146	0.73
Inferior	0.117	0.052~0.285	0.17
Temporal	0.224	0.048~0.400	0.014
**Number of affected subfield (0 as reference)**			
1	0.159	0.064~0.206	0.29
2	−0.039	0.063~0.465	0.835
3	−0.087	0.165~0.106	0.651
4	0.691	0.314~0.751	<0.001

^*^Adjusted by age, sex, preoperative BCVA.

### Layer thickness analysis

**Figure 3:**
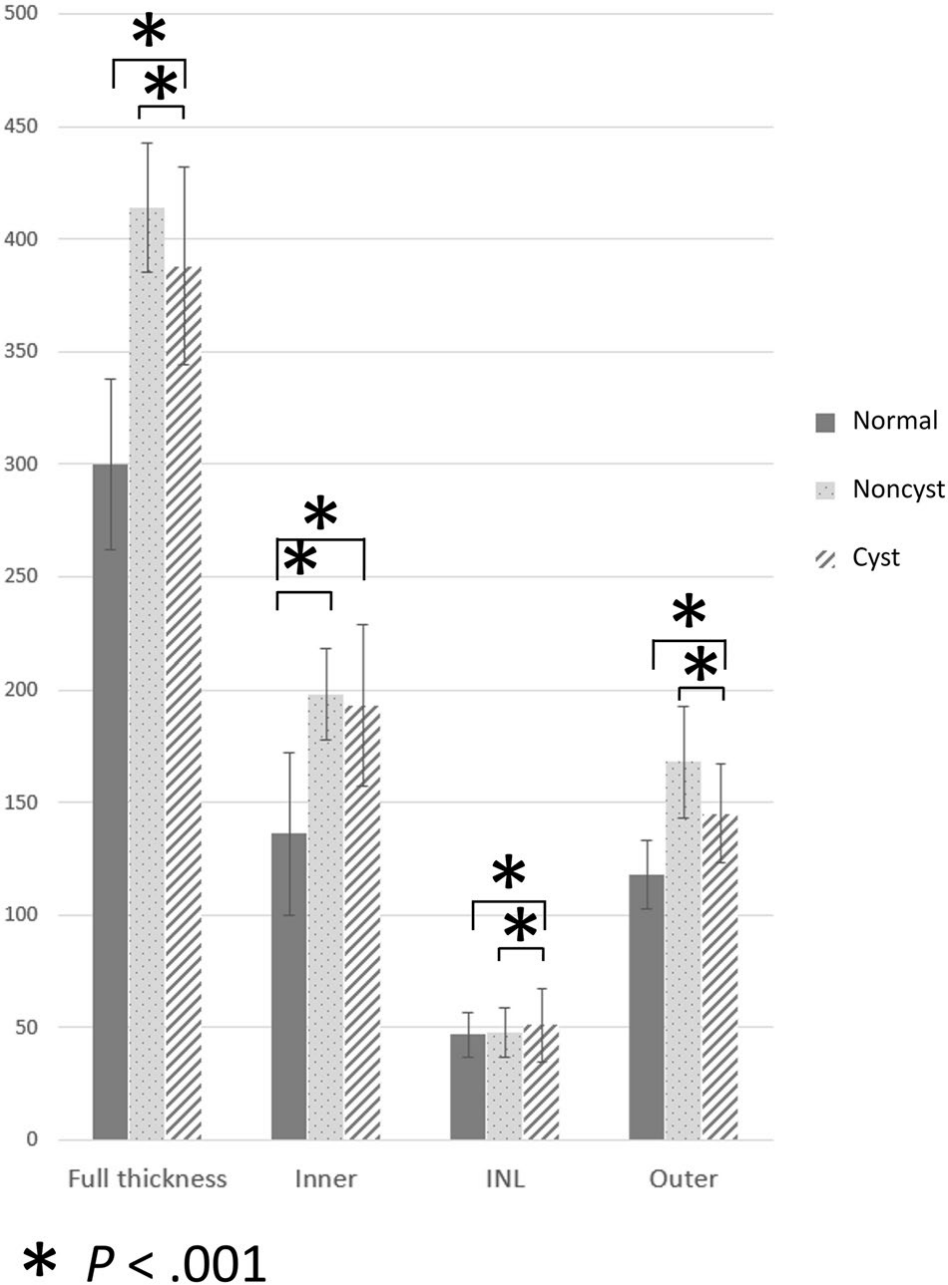
The retinal layer thickness at the nasal subfield of normal control, patients without inner nuclear layer (INL) microcysts (Noncyst), and patients with INL microcysts (Cyst). The inner retinal layer (Inner) measured from the nerve fiber layer to the inner plexiform layer. The outer retinal layer (Outer) includes the outer plexiform layer and the outer nuclear layer to the retinal pigment epithelium. The INL in the microcystic group was thicker than in the nonmicrocystic and normal group groups. The full, inner, and outer retinal layers were both thicker in the patient group than in the normal group. The outer retinal layer in the microcystic group was significantly thinner than in the nonmicrocystic group, whereas the inner retinal layer was not significantly different between the two patient groups.

For retinal layer thickness analysis (Fig. 3), the INL thickness with microcysts (51 ± 16 μm) was thicker than those without microcysts (48 ± 11 μm, *P* < 0.001). However, the total retinal thickness with microcysts (388 ± 44 um) was thinner than those without microcysts (414 ± 29 μm, *P* < 0.001) because of a thinner outer retinal layer in those with microcysts (145 ± 27 vs 168 ± 25 μm, *P* < 0.001).

## Discussion

In this study, by using OCT angiographic mode for en-face imaging, we found that INL microcysts are a common retinal change in patients with ERM after double membrane peeling. Sixty-one percent of patients developed INL microcysts during the follow-up period. This rate was more frequent than in our previous study of 29% that were imaged with only two cross-sectional OCT scans and at a lower resolution. Compared with other studies with even lower incidences, in addition to the more severe preoperative features of our patients, such as poorer presenting visual acuity, thicker macula, or longer follow-up period^5^, the image tools and methods to detect the microcysts were key.

The OCT angiography used 70 000 A-scans per second with an axial resolution of 5 μm and a transverse resolution of 15 μm^7^. A macular examination which contains 180 000 A-scans for an area of 3 × 3 mm could be finished in less than 3 seconds to minimize motion artifacts and acquire higher quality of image. Using a novel angiography algorithm, an application was developed to investigate many small inner retinal lesions and minor changes, such as those in the inner retinal capillary plexus, foveal avascular zone, and peripapillary capillary^8,9^. However, this system still has a considerable advantage even with only the tomography from split-spectrum amplitude-decorrelation angiography algorithms^10,11^. The en face of the tomography, generated from more than 200 B-scans in 3 mm, could ideally provide a 15-μm transverse resolution to evaluate INL microcysts.

The finer resolution OCT used in our study not only showed higher prevalence of microcysts as compared with our previous study, the risk factors and visual outcomes identified also differed from those of the last study. More acute symptoms before surgery, more severe grades of ERM, and intraoperative retinal hemorrhage were no longer the associated factors for postoperative microcysts as observed in this study^5^. Furthermore, the presence of microcysts was not associated with poorer visual outcomes as previously observed. In the previous study, the microcysts were underestimated and their locations were mainly at the center 1 mm and nasal side by 2 cross-sectional OCT. However, wider areas of more than 4 subfields and involvement of center and temporal subfields in this study were still associated with acute symptoms of less than 6 months and poorer functional recovery. This implied that microcysts are a common phenomenon after ERM surgery and that more severe forms of microcysts, such as a wider involved area, are a prognostic factor for worse visual recovery.

Most of the patients had INL microcysts in their nasal subfields. In patients who had microcysts distributed in less than 2 subfields, 10 of 14 (71%) had microcysts in the nasal area, whereas in patients with larger microcystic areas that extended to more than 2 subfields, all had microcysts in the nasal area. The reason why the nasal subfield was affected more than other subfields is unknown.

In patients with optic atrophy, the INL microcysts were also dominantly located at the papillomacular bundle area^12^. The authors proposed that the cell volume loss in the ganglion cell layer and thinning of the nerve fiber layer make the INL expand vertically to compensate for the space created by the vitreous traction, hence the formation of microcysts. Although there was no optic atrophy in our patients and the vitreous was already removed, it was possible that after surgery, the decrease of the ganglion cell layer with the still-stretched and expanded INL might leave space for compensated microcyst formation^13^. The thickness analysis in the nasal subfield (as shown in Fig. 3) confirmed that in regions of microcysts, the INL thickness increased more than those without microcysts. According to this hypothesis, the microcysts should become less when the whole retinal layer thickness decreased following the surgery, yet the microcysts persisted for long as shown in this 16-month study. The other possible mechanism is actual cell loss but without compensated spacing.

Compared with macular holes, Akira Hirata *et al*. found that specimens of peeled ILM after ERM surgery had larger and higher numbers of cell fragments on the retinal side, indicating higher risk of inner retinal damage^14^. However, it was unknown if there were topographic changes to the peeled ILM such that more fragments were found in the nasal side of the ILM (personal discussion with Dr. Akira Hirata). Sigler *et al*. claimed that the INL microcysts had a delayed onset and were the likely result of Müller cell depletion through various intracellular mechanisms after physical trauma^15^. This retrograde trans-synaptic hypothesis was possible and may be secondary to inner retinal loss after ILM peeling. However, it was unknown if the Müller cells distributed differently at the macula so that there were more densely packed Müller cells at the papillomacular bundle, and hence, more cell loss in the nasal subfield. The preponderance of microcysts in the nasal subfield remains speculative.

Our thickness layer analysis in Fig. 3 shows that the INL thickness in areas with microcysts was higher than in patients without microcysts. This might indicate a poorer functional recovery, because the middle retinal layer thickness is negatively correlated with b wave and oscillatory potential amplitude after ERM surgery^16^. However, the full thickness layer in areas with microcysts was thinner than areas without microcysts. This was because of a thinner outer retinal layer underneath the microcysts but not the inner retinal layer thickness above the INL, which was comparable between the 2 (Fig. 3). In an animal model, conditional selectively impaired Müller cells may induce outer nuclear layer cell death and photoreceptor apoptosis and yet a relatively preserved inner retina^17^. The microcysts in the INL layer might indicate Müller cell damage that leads to outer retinal layer loss. This observation requires further validation.

Notably, subclinical cystoid macular edema and Irvine–Gass syndrome can mimic INL microcysts. Some topical agents that may cause macular edema should be used with caution. In the present study, most of the eyes observed (38 of 46) had undergone phacoemulsification during the follow-up period. Only 1 patient had cystoid macular edema, which was resolved after Sub-Tenon’s injection of triamcinolone, although the microcysts from before the cataract surgery persisted after the cystoid edema resolved. No significant change of the microcysts occurred in our longitudinal observation of the patients in the microcystic group. Fluorescein angiography was performed for a suspicious patient, and no dye pooling was found, indicating that the post-vitrectomy microcysts were not related to inflammation or vascular leakage.

This study had some limitations, which included that as a retrospective study, some of the patients might not have had regular OCT evaluations, especially at the early postoperative period. Second, the artifacts of en-face OCT may still obscure the detection of microcysts, especially when the microcysts were small or sparse. In such cases, manual segmentation of OCT images was performed to search the microcysts and eliminate the error during autosegmentation. Third, dividing the macula into five ETDRS areas is a new strategy that has not been validated in ERM studies. We thought that dividing the areas into nasal, intermediate (superior, inferior, and central), and temporal spaces sequentially was a reasonable approach because of the anatomy of the retinal nerve fiber layer.

High-speed scanning OCT could provide acceptable imaging quality to evaluate INL microcysts, which were rarely found using conventional OCT in patients with idiopathic ERM after double membrane peeling. The microcysts appeared as clusters of densely packed spheroidal cystic defects of 80 um in diameter and mostly located at nasal subfield. Although patients with microcysts attained a final visual acuity of greater than 20/28, patients with microcysts at the central and temporal areas and with wider involved areas of greater than 4 subfields had poorer outcomes.

## Methods

We retrospectively reviewed the medical records of patients who had idiopathic ERM and underwent surgery from March 2015 to February 2016. Exclusion criteria included the presence of other retinal disease, secondary ERM, glaucoma, and optic atrophy. Postoperative patients who had transient INL cystic edema following cataract surgery were not enrolled as having INL microcysts. Patients with insufficient OCT images or a follow-up period of less than 12 months were excluded. All the participant have signed and agreed the relevant inform consent. This study was approved by the institutional review board and ethics committees of Taipei Veteran General Hospital in Taiwan. We also confirmed that all research was performed in accordance with the relevant guidelines and regulations.

### Surgical technique

All patients underwent pars plana vitrectomy and double membrane peeling performed by the same surgeon (SJ Chen). The brilliant blue G-assisted membrane peeling was performed as previously described in detail for all cases^5^. All internal limiting membrane at the macular area was peeled, and the initiation site was always selected outside the macula. At the end of the surgery, fluid gas exchange with air was performed, and all patients were instructed to remain in a prone position for 2 days.

All patients underwent comprehensive ophthalmic examination, including best-corrected visual acuity (BCVA), fundus color photographs, and OCT. The OCT was performed by a trained technician with a commercial OCT scanner, the Avanti RTVue XR (OptoVue, Fremont, CA, USA). The “Angio” mode was used, which contains more than 180 000 A-scans in a 3 × 3 mm image volume. The microcysts in the INL were defined by groups of spindle-shaped hyporeflective hollows within the layer in the cross-sectional OCT and appeared as multiple continuous adjacent dark spots by the “en face” of the deep capillary plexus (Fig. 1). The microcysts were revealed after manual adjustment for proper segmentation if needed.

We divided the center 3 × 3 mm into 5 subfields, the superior, nasal, inferior, temporal, and center, for further analysis (Fig. 1).

Multivariate linear regression analysis was used to evaluate the association between the presence of INL microcysts and visual outcome in each area, adjusted by age and preoperative visual acuity. The area of each INL microcyst cluster was drawn manually in each eye (Fig. 1B, irregular circle) and all areas were added on to be presented as a distribution and frequency map after transformation to the left eye configuration by mirror image. To understand the thickness changes in the retinal layers in the nasal subfield of microcysts, 9 patients with microcysts, 3 patients without microcysts, and 4 age-matched normal controls with good image quality were enrolled for comparison. The clustering microcysts were randomly sampled with a maximum of 120 points for each patient. In those patients without microcysts, the sampling consisted of 120 points evenly distributed in the nasal subfield. The thickness of each segmentation of each retinal layer was measured with Orion OCT analysis software (Voxeleron, Pleasanton, CA, USA) that could semi-automatically perform image segmentation and calculate the thickness of the region of interest. The statistic was calculated using analysis of variance, and Scheffe post hoc analysis was performed among the three groups. Regarding the large number of samples *P* = 0.001 was considered as the level of significance.
